# Species-dependent Clinical Findings of Malaria Caused by Various Plasmodia in an Endemic Area of Kerman Province, Southeastern Iran

**Published:** 2017-04

**Authors:** Mehdi NATEGHPOUR, Ali HOSSEININASAB, Mehrdad FARROKHNIA, Foroughieh DASTOURI, Katayun ALIDOOSTI, Dadkhoda SADEQUI, Asadollah AHMADI

**Affiliations:** 1. Dept. of Medical Parasitology and Mycology, School of Public Health, Tehran University of Medical Sciences, Tehran, Iran; 2. Center for Research of Endemic Parasites of Iran, Tehran University of Medical Sciences, Tehran, Iran; 3. Infectious and Tropical Research Center, Kerman University of Medical Sciences, Kerman, Iran; 4. Kerman Province Health Center, Kerman University of Medical Sciences, Kerman, Iran; 5. Kahnouj Health Center, Jiroft University of Medical Sciences, Jiroft, Iran

**Keywords:** Malaria, Clinical finding, Iran

## Abstract

**Background:**

Malaria is a big problem of public health in many tropical countries where socioeconomic development is deficient. Four species of plasmodium are capable of infecting human: *P. falciparum*, *P. malaria*, *P.vivax*, *P. ovale*. Southeastern corner of Iran, including Sistan and Baluchestan, Hormozgan and the tropical part of Kerman Province, are endemic region of malaria. This study aimed to find out clinical findings in malaria caused by various plasmodium species in moderate transmission area of southern Kerman Province.

**Methods:**

This study was conducted in health centers of Kahnooj, Manujan, Ghale-Ganj, Roudbar and Fariab in south of Kerman Province, Southeastern Iran during 2005–2009. Three hundred and thirty patients with positive malaria parasite slides entered the study. Frequencies of several malaria clinical presentations were investigated in four plasmodium species.

**Results:**

54.2% of considered patients were male. Mean age of patients was 22.8±17.8 yr. Younger and older patient were 6 months and 80 yr, respectively. Ten patients were infected with *P. falciparum*, 314 with plasmodium vivax and 6 with mixed infection. The symptoms of fever, chills and sweating were present in 74.6% of subjects. Other complaints were joint pain, headache, fatigue, vomiting, and diarrhea. Splenomegaly was detected in 17.57% of the individuals.

**Conclusion:**

Malaria should be considered in differential diagnosis of all acutely febrile patients in endemic area. Classic symptoms of fever, chills and sweating may not present in all of patients.

## Introduction

Malaria is the most important parasitic infection in the world that poses major health challenges. Despite years of continual efforts, this infection is still a threat to approximately 40% of the world’s population in about 100 countries (1). The disease is caused by plasmodium parasites and transmitted by female anopheles mosquitoes. Four species of malaria parasites cause this disease (*P*. *falciparum*, *P. vivax*, *P. malariae*, *P. ovale)*.

This infection involves many organs and has several manifestations. Its impact varies due to epidemiological settings. Prompt diagnosis and early appropriate treatment, could improve the outcomes of patients and reduce the mortality of malaria infection (2).

Clinical features include fever, chills, sweating, headache, vomiting, diarrhea, abdominal pain, cough, splenomegaly and hepatomegaly (3–5). In non-endemic area, fever is a good sign of malaria in children under 5 yr old (6). Clinical signs and symptoms, routine laboratory test, and travel history are helpful to differentiate of malaria from other febrile diseases (7). Combination of several signs and symptoms has higher positive predictive value to the diagnosis of malaria than each single of them (8). Malaria case definitions using only clinical manifestations are affected by the patient’s age and endemicity of the geographic area (9). Patients from endemic area have significantly better response to drug therapy and prognosis in comparison to the patients from low malaria transmission regions (10). Asymptomatic malaria in endemic area is a big problem for malaria control program in these regions (11). Southeastern corner of Iran including Sistan and Baluchestanand, Hormozgan, and the tropical part of Kerman province are endemic region of malaria (12). In Iran, malaria is remaining as a public health problem (13). The main objective of this study was to determine the common clinical features in malaria caused by various Plasmodium species in south of Kerman province.

## Materials and Methods

This study was conducted in health centers of Kahnooj, Manujan, Ghale-Ganj, Roudbar and Fariab districts in south of Kerman Province, Southeastern Iran during 2005–2009. After obtaining informed consent, three hundred and thirty individuals with malaria infection were included in the study.

Giemsa stained thick and thin blood smear were made from all of the subjects and examined according to the Basic Malaria Microscopy guideline (14). Clinical evaluation including fever, chills, sweating, vomiting, headache, diarrhea, abdominal pain, cough, herpes labialis, splenomegaly, and hepatomegaly were recorded. The collected data were analyzed through SPSS19 software (Chicago, IL, ISA). Some quantitative and qualitative variables including central distribution indices, frequencies of various symptoms and signs and percentages were determined.

## Results

Overall, 179(54.2%) and 151(45%) were male and female, respectively. Mean age of patients was 22.8±17.8 yr old. Younger and older patient were 6 months and 80 yr, respectively. Ten patients were infected with *Plasmodium falciparum*, 314 of them with *Plasmodium vivax* and the rest had mixed infection of *P. vivax* and *P. falciparum* (Table 1). Classic triad of fever, chills and sweating were the leading clinical presentations in the three forms of infections and this triad was found in 80.84% of *P. vivax* and 50% of mixed infections while just in10% of the subjects with *P. falciparum*. Fever was the most symptoms in majority of the patients. Splenomegaly was the leading sign ranging from two to 23%. Herpes labialis was found in both *falciparum* and *vivax* infections ranging from 1% to 3%. This finding was not observed in mixed infections. Other main symptoms and sings were headache, chills, sweating, joint and bone pain, fatigue, hepatomegaly vomiting and diarrhea. Totally, 98% of the patients were treated as outpatient without any complication. More details are shown in Table 2.

**Table 1:** Various plasmodia species infection based on age and gender of patients

**Plasmodia Parameters**		***P. falciparum* (%)**	***P. vivax* (%)**	Mixed^*^ (%)	**Total (%)**
		**n=10(3.03)**	**n=314(95.15)**	**n=6(1.81)**	**n=330(100)**
Gender	Male	5(50)	171(54.45)	5(83.33)	181(54.84)
	Female	5(50)	143(45.54)	1(6.77)	149(45.15)
Range of ages yr		2–78	0.5–80	1–53	0.5–80
(Mean)		(23.2)	(22.8)	(23.9)	(22.8)

* Mixed: *P. falciparum*+*P. vivax*

**Table 2:** Clinical findings in various plasmodia species infections

**Plasmodia Symptoms**	***P. falciparum* n (%)**	***P. vivax* n (%)**	***Mixed* n (%)**	**Total n (%)**
Fever	7(70)	314(100)	6(100)	327(99.09)
Fatigue	1(10)	79(25.15)	1(16.66)	81(24.54)
Chills	6(60)	295(93.94)	3(50)	304(92.12)
Sweating	1(10)	251(79.93)	4(66.66)	256(77.57)
Classic triad*	1(10)	173(80.84)	3(50)	177(53.63)
Headache	3(30)	93(29.61)	5(83.33)	101(30.60)
Bone pain	3(30)	228(72.61)	4(66.66)	235(71.21)
Vomiting	1(10)	49(15.60)	2(33.33)	52(15.75)
Abdominal pain	0(0)	28(8.91)	0(0)	28(8.48)
Diarrhea	0(0)	28(8.91)	0(0)	28(8.48)
Cough	2(20)	31(9.87)	0(0)	33(10)
Pallor	0(0)	20(6.36)	1(16.66)	21(6.36)
Herpes labialis	1(10)	3(0.95)	0(0)	4(1.21)
Splenomegaly	0(0)	57(18.15)	1(16.66)	58(17.57)
Hepatomegaly	0(0)	17(5.41)	0(0)	17(5.15)
Jaundice	0(0)	20(6.36)	0(0)	20(6.06)
Hospitalized	3(30)	2(0.64)	1(16.67)	6(1.81)

* Chills, fever and sweating

## Discussion

This study tried to elucidate the main clinical species-dependent symptoms of malaria infection based on the native strain of the parasites. In this study, almost all patients (98%) had uncomplicated malaria presented by fever, chills, headache, sweating, myalgia and joint pain such one other study that designed in Colombia (15) Pakistan and (16). Those results released by a number of authors from Zimbabwe, Thailand and some parts of Pakistan were comparable with those obtained from this study about *P. falciparum* symptoms (5, 17, 18). The main three signs in *P. vivax* of this study were the same with others (19). Vomiting was noted in 15% of patients infected with *P. vivax*and in 10% of *P. falciparum* infection that was significantly less than those were reported from Thailand with 37% of the infections (18).

Herpes labialis was found in both *falciparum* and *vivax* but not in mixed infection in this study similar to those results found others (16). In another study, this sign is reported only in falciparum cases (20).

Splenomegaly was recorded in 57% and 16% of patients with *P.vivax* and mixed infection, respectively. None of the *P. falciparum* infected patients had splenomegaly. This high proportion of splenomegaly in Kerman province is contrary to those reported from Quetta (5). Such discrepancy may explain due to repeat *vivax* malaria infection occurred among the studied patients. Mild splenomegaly was mostly reported with *P. vivax* (15). Hepatomegaly rate of *P. vivax* detected in our study was similar to those reported from Colombia (19), but dissimilar to those were in Thailand (18). This study highlighted some different clinical presentation due to different plasmodium species. Moreover, more study is required to show the relationship between clinical presentation and plasmodium species.

## Conclusion

Clinical presentations of various plasmodia species are almost similar in the most malarious areas in the world, but with some differences. In our study patients with *vivax* malaria infection showed more classic clinical symptoms than either *falciparum* malaria or mixed infected patients.

## Ethical considerations

Ethical issues (Including plagiarism, informed consent, misconduct, data fabrication and/or falsification, double publication and/or submission, redundancy, etc.) have been completely observed by the authors.

## References

[1] Na-Bangchang K Congpuong K (2007). Current malaria status and distribution of drug resistance in East and Southeast Asia with special focus to Thailand. Tohoku J Exp Med, 211:99–113. 17287593 10.1620/tjem.211.99

[2] Sagaki P Thanachartwet V Desakorn V Sahassananda D Chamnanchanunt S Chierakul W Pitisuttithum P Ruangkanchanasetr P (2013). Clinical factors for severity of Plasmodium falciparum malaria in hospitalized adults in Thailand. PLoS One, 8:e71503. 23951178 10.1371/journal.pone.0071503 PMC3741184

[3] Malik GM Seidi O El-Taher A Mohammed AS (1998). Clinical aspects of malaria in the Asir Region, Saudi Arabia. Ann Saudi Med, 18:15–7. 17341908 10.5144/0256-4947.1998.15

[4] Hozhabri S Akhtar S Rahbar MH Luby SP (2000). Prevalence of plasmodium slide positivity among the children treated for malaria, Jhangara, Sindh. J Pak Med Assoc, 50:401–5. 11191438

[5] Sheikh AS Sheikh AA Sheikh AS Paracha SM (2005). Endemicity of malaria in Quetta. Pakistan. J Med Res, 44:1–5.

[6] Mutanda AL Cheruiyot P Hodges JS Ayodo G Odero W John CC (2014). Sensitivity of fever for diagnosis of clinical malaria in a Kenyan area of unstable, low malaria transmission. Malar J, 13:163. 24885660 10.1186/1475-2875-13-163 PMC4021053

[7] Kutsuna S Hayakawa K Kato Y Fujiya Y Mawatari M Takeshita N Kanagawa S Ohmagari N (2015). Comparison of clinical characteristics and laboratory findings of malaria, dengue, and enteric fever in returning travelers: 8-year experience at a referral center in Tokyo, Japan. J Infect Chemother, 21:272–6. 25592811 10.1016/j.jiac.2014.12.004

[8] Feleke SM Animut A Belay M (2015). Prevalence of malaria among acute febrile patients clinically suspected of having malaria in the Zeway Health Center, Ethiopia. Jpn J Infect Dis, 68:55–9. 25420658 10.7883/yoken.JJID.2013.062

[9] Afrane YA Zhou G Githeko AK Yan G (2014). Clinical malaria case definition and malaria attributable fraction in the highlands of western Kenya. Malar J, 13:405. 25318705 10.1186/1475-2875-13-405 PMC4209040

[10] Ziaee M Abedi F (2014). Severe falciparum malaria in iran: a very rare case from an endemic region. Jundishapur J Microbiol, 7:e8752. 25147660 10.5812/jjm.8752 PMC4138670

[11] Singh R Godson II Singh S Singh RB Isyaku NT Ebere UV (2014). High prevalence of asymptomatic malaria in apparently healthy schoolchildren in Aliero, Kebbi state, Nigeria. J Vector Borne Dis, 51:128–32. 24947220

[12] Zayeri F Salehi M Pirhosseini H (2011). Geographical mapping and Bayesian spatial modeling of malaria incidence in Sistan and Baluchistan province, Iran. Asian Pac J Trop Med, 4:985–92. 22118036 10.1016/S1995-7645(11)60231-9

[13] Raiesi A Nikpour F Ansari-Moghaddam A Ranjbar M (2011). Baseline results of the first malaria indicator survey in Iran at the health facility level. Malar J, 10:319. 22029447 10.1186/1475-2875-10-319 PMC3213221

[14] WHO (1991). Basic Malaria Microscopy Part 1 Learner’s Guide, Part 2 Trainer’s Guide. Geneva. http://apps.who.int/iris/bitstream/10665/44208/1/9789241547826_eng.pdf

[15] Arevalo-Herrera M Lopez-Perez M Medina L Moreno A Gutierrez JB Herrera S (2015). Clinical profile of Plasmodium falciparum and Plasmodium vivax infections in low and unstable malaria transmission settings of Colombia. Malar J, 14:154. 25889074 10.1186/s12936-015-0678-3 PMC4397685

[16] Rasheed A Saeed S Khan SA (2009). Clinical and laboratory findings in acute malaria caused by various plasmodium species. J Pak Med Assoc, 59:220–3. 19402282

[17] Stein CM Gelfand M (1985). The clinical features and laboratory findings in acute Plasmodium falciparum malaria in Harare, Zimbabwe. Cent Afr J Med, 31:166–70. 3910259

[18] Erhart LM Yingyuen K Chuanak N Buathong N Laoboonchai A Miller RS Meshnick SR Gasser RA Jr. Wongsrichanalai C (2004). Hematologic and clinical indices of malaria in a semi-immune population of western Thailand. Am J Trop Med Hyg, 70:8–14. 14971691

[19] Echeverri M Tobon A Alvarez G Carmona J Blair S (2003). Clinical and laboratory findings of Plasmodium vivax malaria in Colombia, 2001. Rev Inst Med Trop Sao Paulo, 45:29–34. 12751319 10.1590/s0036-46652003000100006

[20] Sowunmi A Gbotosho GO Adedeji AA Tambo E Bolaji OM Happi CT Fateye BA ( 2008 ). Herpes simplex labialis in children with acute falciparum malaria . Acta Trop, 106 : 68 –71. 18313630 10.1016/j.actatropica.2007.03.005

